# The advanced lung cancer inflammation index as a predictor of kidney stone risk in men: a cross-sectional analysis

**DOI:** 10.3389/fnut.2025.1568427

**Published:** 2025-07-24

**Authors:** Yan Zhou, Xiaomin Li, Qiying He, Qin Feng, Yu Liu, Banghua Liao

**Affiliations:** ^1^Department of Urology, Institute of Urology, West China Hospital, Sichuan University, Chengdu, China; ^2^West China School of Nursing, Sichuan University, Chengdu, China; ^3^Institute of Transfusion Medicine and Immunology, Medical Faculty Mannheim, Heidelberg University, Heidelberg, Germany; ^4^Department of Urology and Institute of Urology (Laboratory of Reconstructive Urology), West China Hospital, Sichuan University, Chengdu, Sichuan, China

**Keywords:** advanced lung cancer inflammation index, kidney stone, inflammation, nutrition, National Health and Nutrition Examination Survey (NHANES) obesity

## Abstract

**Background:**

Kidney stone incidence and recurrence are increasing, which poses significant health problems. The Advanced Lung Cancer Inflammation Index (ALI) combines body mass index (BMI), albumin, and neutrophil-to-lymphocyte ratio (NLR). It was first created to predict outcomes for cancer patients. Recently, it has been studied as an indicator of overall inflammation and nutrition in other diseases, such as chronic kidney disease, heart failure, acute myocardial infarction, and asthma. This study aimed to evaluate the association between ALI scores and kidney stone risk in adult men, and to assess the potential utility of ALI as an indicative biomarker.

**Methods:**

We analyzed data from 5,429 male participants in the National Health and Nutrition Examination Survey (NHANES, 2007–2018). Weighted logistic regression models were used to examine the relationship between ALI scores and the risk of kidney stones. Non-linear associations were further explored with smoothed curve fitting. The predictive value of ALI was assessed using receiver operating characteristic (ROC) curve analysis. Subgroup analyses were conducted to evaluate the consistency of findings across different demographic and clinical characteristics.

**Results:**

Out of all participants, 572 (10.5%) had a history of kidney stones. For every one-unit increase in ALI score, the risk of having kidney stones decreased by 22.7% (odds ratio = 0.773, 95% CI: 0.675–0.885, *P* < 0.001). The smooth curve analysis showed a non-linear inverse relationship. The protective effect was stronger when ALI scores were low. Subgroup analyses showed stronger relationships for men aged 60–80, overweight men, former smokers, and men without hypertension, diabetes, or cardiovascular disease. ROC analysis showed ALI had moderate accuracy in predicting kidney stones (AUC = 0.770).

**Conclusion:**

ALI scores were independently linked to a lower risk of kidney stones, especially in men without metabolic diseases. As a simple inflammation and nutrition marker, ALI could help identify people who have a higher risk. However, due to the cross-sectional design of this study, a causal relationship between ALI and kidney stone risk cannot be established. Further prospective studies are needed to validate these findings.

## Introduction

Kidney stone disease is a common urological disorder defined by the formation of crystalline deposits within the kidneys. In recent decades, its incidence has increased globally, with a prevalence now exceeding 10% and recurrence rates above 50% in the United States (1, 2). The causes of KSD are complex and include metabolic, dietary, genetic, and environmental factors. KSD is now increasingly viewed as a chronic metabolic condition, closely linked to obesity, diabetes, and unhealthy eating habits (3, 4). In addition to acute symptoms, KSD increases the risk of urinary tract infection, chronic kidney disease, and hypertension, and poses a significant burden on healthcare systems (5).

Among the many factors that contribute to the development of KSD, systemic inflammation and nutritional status are thought to be especially important. Long-term mild inflammation is linked to many metabolic diseases and might lead to kidney stones by changing urine composition, encouraging crystal growth, and reducing kidney function (6–9). Similarly, poor nutritional status can influence urinary pH and the excretion of stone-forming elements such as calcium and uric acid, further increasing the risk of stone formation (10, 11).

The Advanced Lung Cancer Inflammation Index (ALI) is a measure that combines body mass index (BMI), serum albumin levels, and neutrophil-to-lymphocyte ratio (NLR) to show overall inflammation and nutritional status. It was first used to predict outcomes for lung cancer patients (12), but it is now also used to assess health conditions such as heart disease, diabetes, and chronic kidney disease (13–16). Since both inflammation and poor nutrition are linked to kidney stones, ALI might be a useful way to predict who is at risk of kidney stones.

However, to date, the association between ALI and kidney stone risk has not been systematically investigated in a general population. This study used data from the National Health and Nutrition Examination Survey (NHANES) to find out if ALI is related to the risk of having kidney stones. Understanding the relationship between ALI and kidney stones could help explain how inflammation and nutrition affect stone formation and improve methods for predicting risk.

## Materials and methods

### Study design and setting

This study was a cross-sectional analysis utilizing data from NHANES, a nationally representative program designed to assess the health and nutritional status of the U.S. population. NHANES uses a sampling method with several steps to select participants. It collects information through interviews, physical exams, and lab tests. Data from six NHANES cycles (2007–2018) were included in the present analysis.

### Sample size justification

Initially, 59,842 participants were selected from NHANES data. After applying eligibility criteria, 5,429 adult male participants were included in the final analysis. The sample size depended on the availability of complete information about ALI and kidney stones. No extra sample size calculation was done since we used all available NHANES data.

### Eligibility criteria

**FIGURE 1:**
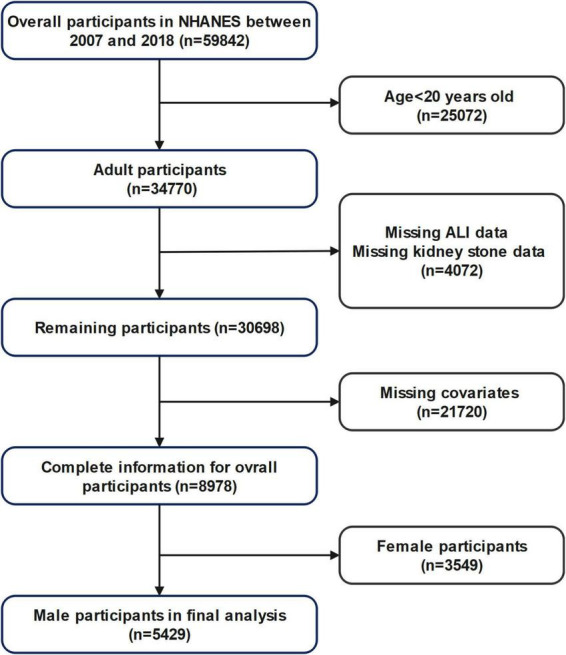
Diagram illustrating the process of selecting participants.

Inclusion criteria: (1) Male participants aged 20 years or older; (2) Availability of complete data for ALI calculation (including BMI, serum albumin, neutrophil, and lymphocyte counts); (3) Availability of self-reported kidney stone history. Exclusion criteria: (1) Age < 20 years; (2) Missing data on ALI, kidney stone status, or relevant covariates; (3) Female participants. A detailed flowchart of participant selection is provided in Figure 1.

### Exposure and outcome definitions

The exposure variable was ALI, calculated as: ALI = serum albumin (g/dL) × BMI (kg/m^2^)/NLR. Serum albumin and BMI were obtained from laboratory and physical examination data, while NLR was calculated from complete blood count results. ALI values were divided into quartiles: Q1 (2.89–43.99), Q2 (44.00–60.94), Q3 (60.95–83.27), and Q4 (≥83.28).

The primary outcome was the self-reported history of kidney stones, ascertained from the questionnaire item: “Have you ever had a kidney stone?” (Variable ID: KIQ026). Participants answering “yes” were classified as having a history of kidney stones; those answering “no” were considered as never having had kidney stones.

### Data collection

Data regarding demographics, socioeconomic status, laboratory findings, and health-related behaviors were extracted from publicly available NHANES datasets (2007–2018). Demographic variables, including age, race, educational level, and poverty-to-income ratio (PIR), were directly retrieved from the NHANES database.

Health behaviors and comorbid conditions were assessed via structured questionnaires. Smoking status was categorized into current smokers (participants reporting current cigarette use, SMQ040) and past smokers (those who smoked at least 100 cigarettes in their lifetime but currently do not smoke, SMQ020). Alcohol drinking history was assessed using specific questionnaire items across survey cycles; from 2007 to 2016, alcohol consumption frequency was derived from questions ALQ120Q (frequency of alcohol consumption in the past 12 months) and ALQ120U (number of drinking days per week/month/year), whereas from 2017 to 2018, this was assessed using question ALQ121 (frequency of alcohol consumption in the past 12 months).

Hypertension was defined based on a self-reported physician diagnosis (BPQ020), self-reported use of antihypertensive medication (BPQ040A), or measured systolic and/or diastolic blood pressure ≥ 140/90 mmHg. Blood pressure was determined by calculating the average of the first three systolic (BPXSY1, BPXSY2, BPXSY3) and diastolic readings (BPXDI1, BPXDI2, BPXDI3).

Diabetes mellitus was identified by self-reported physician diagnosis (DIQ010), current use of antidiabetic medications (DIQ070), or hemoglobin A1c (HbA1c) level ≥ 6.5% (DIQ280).

Cardiovascular disease was determined based on a self-reported history of stroke (MCQ160F), myocardial infarction (MCQ160E), angina pectoris (MCQ160D), coronary artery disease (MCQ160C), or congestive heart failure (MCQ160B).

Laboratory measurements, including serum albumin (g/dL), neutrophils (10^9^/L), lymphocytes (10^9^/L), followed standardized protocols described in detail in the NHANES Laboratory Procedures Manuals. Body mass index (BMI, kg/m^2^) was directly obtained from the NHANES database as a pre-calculated variable based on standardized anthropometric measurements.

### Ethical considerations

The NHANES protocol was approved by the National Center for Health Statistics (NCHS) Research Ethics Review Board under protocol numbers #2005-06 (for cycles 2005–2010), #2011-17 (for cycles 2011–2016), and #2018-01 (for cycles 2017–2018). Further details regarding the ethical approval process are available at the CDC website: https://www.cdc.gov/nchs/nhanes/about/erb.html. All participants provided written informed consent prior to participation. The present analysis used de-identified, publicly available data and was exempt from additional institutional review.

### Statistical analysis

All statistical analyses accounted for NHANES complex, multistage sampling design and sampling weights according to CDC guidelines. Continuous variables were first assessed for normality using the Shapiro–Wilk test; as all showed non-normal distributions, they are presented as survey-weighted medians (interquartile ranges) and compared between groups with the survey-weighted Mann–Whitney U test. Categorical variables are expressed as frequencies (weighted percentages) and compared between groups using the Rao–Scott χ^2^ test.

Weighted logistic regression models were employed to explore the association between ALI and kidney stone prevalence. Results were shown as odds ratios (ORs) with 95% confidence intervals (CIs). ORs were preferred due to their standard application in cross-sectional studies with binary outcomes using complex survey data and multiple covariates. Given the relatively low kidney stone prevalence (10.5%), ORs provide a suitable approximation of risk ratios (RRs), consistent with prior NHANES studies (17, 18).

Three regression models were constructed: Model 1 (unadjusted), Model 2 (adjusted for age, race/ethnicity, education, and PIR), and Model 3 (fully adjusted model including age, race/ethnicity, education, PIR, smoking status, alcohol use, hypertension, diabetes, and cardiovascular disease). Restricted cubic spline analysis was conducted to assess potential non-linear associations between ALI and kidney stones. Subgroup analyses and interaction tests were performed, with Bonferroni correction applied to adjust for multiple comparisons.

Receiver operating characteristic analysis was used to assess ALI’s predictive capability for kidney stones, and the area under the curve (AUC) with 95% CIs was reported to quantify discrimination performance.

Data management and descriptive statistics were performed using SPSS version 26.0 (IBM Corp., Armonk, NY, USA), while logistic regression, spline fitting, ROC analyses, and subgroup analyses were conducted using R version 4.4.1 (R Foundation for Statistical Computing, Vienna, Austria). Two-tailed *P*-values < 0.05 indicated statistical significance. Reporting followed the STROBE guidelines (19).

## Results

### Population characteristics

Adhering to the selection standards shown in Figure 1, the research involved 5429 male subjects from the NHANES database (2007–2018). Among them, 572 (10.5%) had a documented history of kidney stones, whereas 4857 had no kidney stones. Table 1 summarizes the demographic and clinical characteristics according to kidney stone status.

**TABLE 1:** Baseline characteristics of male participants with and without kidney stones: National Health and Nutrition Examination Survey (NHANES) 2007–2018, weighted.

Variable	Overall (*N* = 5429)	Non-kidney stone (*N* = 4857)	Kidney stone (*N* = 572)	*P*-value
Age (years), median (IQR)	47 (34–60)	47 (33–59)	53 (40–65)	**<0.001**
Race (n, %)				**<0.001**
Mexican American	777 (8.4)	716 (8.7)	61 (5.2)	
Other Hispanic	493 (5.1)	428 (5.1)	65 (5.4)	
Non-Hispanic White	2588 (70.8)	2240 (69.8)	348 (78.8)	
Non-Hispanic Black	1034 (8.7)	976 (9.4)	58 (3.7)	
Other race	537 (7.0)	497 (7.0)	40 (6.9)	
Educational level (n, %)				**<0.001**
Lower than 12th grade	1392 (17.7)	1256 (17.7)	136 (17.6)	
High school grade	1485 (27.8)	1350 (28.3)	135 (23.9)	
College grade	2552 (54.5)	2251 (54.0)	301 (58.5)	
PIR (n, %)				**<0.001**
<1.3	1161 (15.4)	1066 (15.9)	95 (11.4)	
≥1.3, <3.5	1363 (26.0)	1189 (25.1)	174 (33.7)	
≥3.5	2905 (58.6)	2602 (59.0)	303 (55.0)	
Smoking history (n, %)				**<0.001**
Current	2533 (44.0)	2323 (45.0)	210 (36.3)	
Past	2896 (56.0)	2534 (55.0)	362 (63.7)	
Alcohol drinking history (drinks/week) (n, %)				**<0.001**
<1	2407 (41.0)	2108 (40.4)	299 (46.0)	
1–4	2090 (40.7)	1910 (40.9)	180 (39.4)	
>4	932 (18.2)	839 (18.7)	93 (14.6)	
Hypertension (n, %)				**<0.001**
Yes	2043 (34.7)	1764 (33.4)	279 (45.6)	
No	3386 (65.3)	3093 (66.6)	293 (54.4)	
Diabetes (n, %)				**<0.001**
Yes	742 (10.7)	612 (9.9)	130 (16.9)	
No	4687 (89.3)	4245 (90.1)	442 (83.1)	
CVD (n, %)				**<0.001**
Yes	741 (10.9)	616 (10.0)	125 (18.3)	
No	4688 (89.1)	4241 (90.0)	447 (81.7)	
BMI (kg/m^2^), median (IQR)	27.80 (24.20–32.40)	27.62 (24.03–32.20)	29.30 (25.50–33.60)	**<0.001**
SAL (g/dL), median (IQR)	4.30 (4.10-4.50)	4.30 (4.10-4.50)	4.20 (4.00–4.40)	**<0.001**
Neutrophils (10^9^/L), median (IQR)	4.20 (3.30–5.40)	4.20 (3.30–5.40)	4.30 (3.40–5.40)	**<0.001**
Lymphocytes (10^9^/L), median (IQR)	2.10 (1.70–2.60)	2.10 (1.70–2.60)	2.10 (1.70–2.50)	**<0.001**
NLR, median (IQR)	2.00 (1.53–2.67)	2.00 (1.52–2.65)	2.05 (1.58–2.80)	**<0.001**
ALI, median (IQR)	60.89 (44.47–80.87)	60.88 (44.43–81.11)	61.00 (44.94–79.45)	**0.001**

Continuous variables are presented as weighted means with standard deviations (SD), and categorical variables are expressed as numbers and weighted percentages. Survey-weighted mean analysis and chi-squared test were used for statistical analysis. PIR, poverty income ratio; CVD, cardiovascular disease; BMI, body mass index; SAL, serum albumin level; NLR, neutrophil to lymphocyte ratio; ALI, advanced lung cancer inflammation index. Bold values in the table body indicate statistical significance at *P* < 0.05.

Men with kidney stones were predominantly Non-Hispanic White and were significantly older. Additionally, these participants had higher socioeconomic status, as indicated by higher educational level and PIR. Notably, they also had significantly lower prevalence of hypertension, diabetes, cardiovascular diseases, and lower rates of smoking and alcohol consumption compared to those without kidney stones (all *P* < 0.001).

Regarding laboratory and clinical parameters, kidney stone participants had significantly higher BMI [29.30 (25.50–33.60) vs 27.62 (24.03–32.20)], neutrophil counts, and NLR [2.05 (1.58–2.80) vs 2.00 (1.52–2.65)], and significantly lower serum albumin [4.20 (4.00–4.40) vs 4.30 (4.10–4.50)], lymphocyte counts, and ALI [61.00 (44.94–79.45) vs 60.88 (44.43–81.11)] (all *P* ≤ 0.001).

### Association between ALI and kidney stone risk

Table 2 demonstrates the link between ALI and the chances of kidney stones in men, as shown by weighted logistic regression analyses. Three separate logistic regression models were established: model 1 didn’t consider all potential confounders, model 2 only accounted for certain factors like age, race, education, and PIR, and model 3 thoroughly examined the following confounders including age, race, education level, PIR, diabetes, drinking status, smoking status, hypertension, CVD. Our ALI analysis covered both continuous and categorical elements (quartiles) to explore its relationship with kidney stones in male subjects. Model 3 highlighted a significant inverse correlation between ALI as a continuous indicator and the probability at onset of kidney stones (*P* < 0.001). Remarkably, an ALI increase of one unit was related to a 22.7% reduction in the risk of kidney stones (OR = 0.773, 95% CI: 0.675–0.885). For ALI categorized into quartiles, Q2 showed a significantly lower risk of kidney stones compared to Q1 (OR = 0.544, 95% CI: 0.315–0.942, *P* = 0.030), indicating that individuals in the Q2 group had 45.6% of the kidney stone risk observed in the Q1 group. However, no statistically significant association was observed for Q3 (OR = 0.488, 95% CI: 0.221–1.082, *P* = 0.077) or Q4 (OR = 1.120, 95% CI: 0.123–10.318, *P* = 0.917).

**TABLE 2:** Weighted logistic analysis of Advanced Lung Cancer Inflammation Index (ALI) and kidney stone risk in males.

Model	ALI (Continuous variable), OR (95% CI), *P* value	Quartile 1 (Reference)	Quartile 2, OR (95% CI), *P* value	Quartile 3, OR (95% CI), *P* value	Quartile 4, OR (95% CI), *P* value
Model 1	1.087 (1.050–1.125), ***P* < 0.001**	Reference	1.426 (0.943–2.156), *P* = 0.092	2.535 (1.667–3.856), ***P* < 0.001**	3.452 (0.716–16.639), *P* = 0.123
Model 2	1.035 (0.992–1.080), *P* = 0.108	Reference	0.866 (0.558–1.343), *P* = 0.520	1.340 (0.860–2.089), *P* = 0.196	3.533 (0.980–12.738), *P* = 0.054
Model 3	0.773 (0.675–0.885), ***P* < 0.001**	Reference	0.544 (0.315–0.942), ***P* = 0.030**	0.488 (0.221–1.082), *P* = 0.077	1.120 (0.123–10.318), *P* = 0.917

OR, odds ratio; 95% CI, 95% confidence interval. Model 1, no covariates were adjusted. Model 2, adjusted for age, race, educational level, and PIR. Model 3, adjusted for age, race, educational level, PIR, smoking history, alcohol drinking history, hypertension, diabetes, cardiovascular disease. Bold values in the table body indicate statistical significance at *P* < 0.05.

**FIGURE 2:**
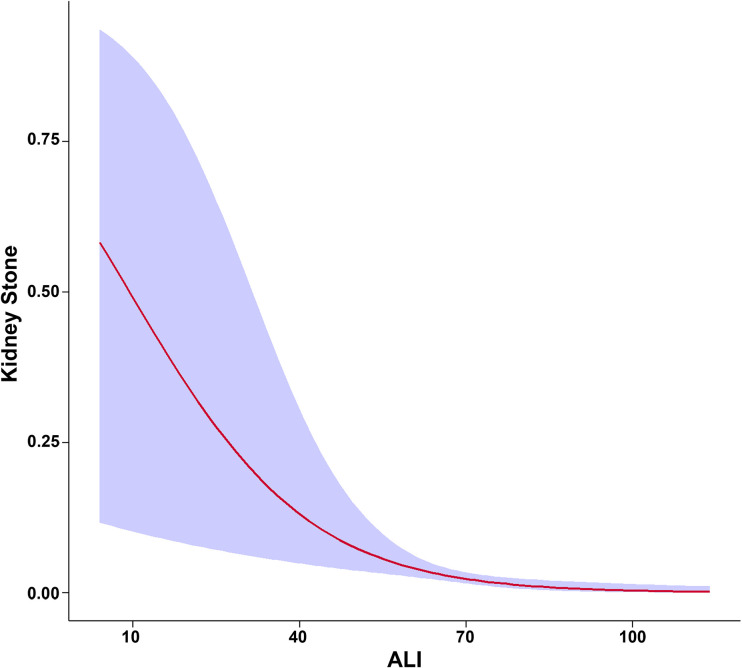
Smooth curve modeling of the association between Advanced Lung Cancer Inflammation Index (ALI) and kidney stone risk in male participants. The red line represents the smooth curve fit between the variables, while the shaded blue area indicates the 95% confidence interval of the fit.

Figure 2 illustrates the smooth curves depicting the relationship between male continuous ALI and the predicted probabilities of kidney stone risk. This curve displays a non-linear reverse trend, where risk decreases sharply with lower ALI levels and stabilizes, without a distinct shift point. At lower ALI values, the odds ratio’s 95% confidence range indicates more variability, decreasing as ALI increases, eventually reaching a horizontal level near *y* = 0. This trend matches Table 2’s outcomes for model 3, demonstrating a significant risk reduction for kidney stones in the Q2 group compared to Q1, based on quartile analysis (*P* < 0.05), while no statistically significant associations were observed in Q3 and Q4. The findings underscore ALI’s non-linear, gradual protective effect in reducing the risk of kidney stones, especially at lower ALI levels.

**FIGURE 3:**
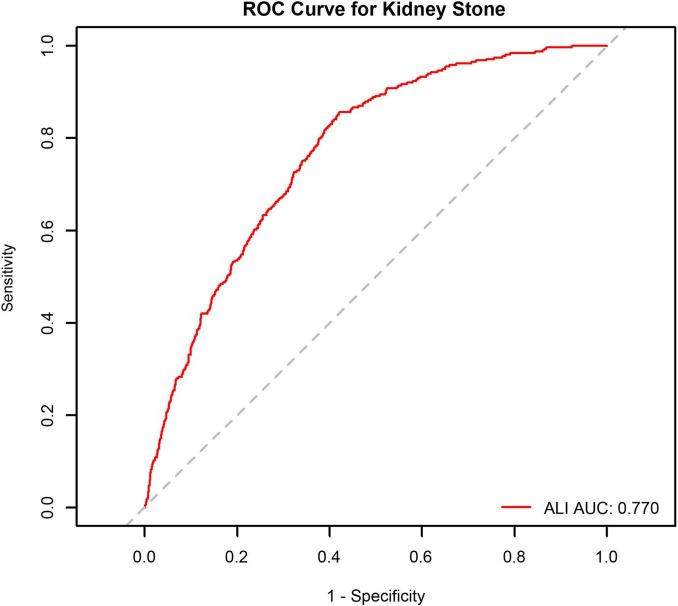
Receiver operating characteristic (ROC) curve analysis of Advanced Lung Cancer Inflammation Index (ALI) for kidney stone occurrence in male participants.

To evaluate the prediction of ALI in kidney stone cases, the ROC curve analysis (see Figure 3) was employed (AUC = 0.770, 95% CI: 0.747–0.794).

Given the observed association between ALI and kidney stone risk in males, we further explored whether a similar relationship existed in females. A separate sex-stratified analysis was performed to assess potential gender-specific effects. The results, presented in Supplementary Figures 1, 2 and Supplementary Table 1, demonstrated that ALI was not significantly associated with kidney stone risk in the female subgroup after full adjustment (Model 3: OR = 1.020, 95% CI: 0.944–1.101, *P* = 0.616). Notably, the smoothed curve analysis indicated a mild upward trend between ALI and kidney stone risk in females, which was in contrast to the inverse association observed in males. In addition, ROC curve analysis revealed a relatively low AUC value of 0.633, suggesting that ALI has limited discriminatory ability in predicting kidney stones among females. These findings collectively underscore a possible sex-specific difference in the predictive relevance of ALI, potentially influenced by biological or hormonal factors.

### Subgroup analysis

Subgroup analyses were conducted to assess potential effect modification in the association between ALI and kidney stone risk (Table 3). A significant inverse association was observed among participants aged 60–80 years, where each unit increase in ALI was associated with a 28.2% reduced risk of kidney stone (OR = 0.718, 95% CI: 0.579–0.890, *P* = 0.003). A similar protective effect was identified in the overweight group (BMI ≥ 25 and <30), with an OR of 0.721 (95% CI: 0.584–0.891, *P* = 0.002).

**TABLE 3:** Weighted subgroup analysis with full adjustment for the association between Advanced Lung Cancer Inflammation Index (ALI) and kidney stone risk in male participants.

Subgroup	ALI (OR, 95% CI)	*P*-value	*P* for interaction
Age			0.019
20–39	0.789 (0.558–1.116)	0.181	
40–59	0.822 (0.654–1.033)	0.094	
60–80	0.718 (0.579–0.890)	**0.003**	
Race			0.218
Mexican American	0.711 (0.524–0.965)	0.029	
Other Hispanic	1.090 (0.630–1.888)	0.758	
Non-Hispanic White	0.751 (0.617–0.915)	**0.004**	
Non-Hispanic Black	0.922 (0.766–1.110)	0.393	
Other race	0.954 (0.594–1.533)	0.846	
Educational level			0.188
Lower than 12th grade	0.840 (0.653–1.079)	0.172	
High school grade	0.857 (0.666–1.103)	0.232	
College grade	0.716 (0.595–0.861)	**0.001**	
PIR			0.509
<1.3	0.829 (0.671–1.024)	0.082	
≥1.3, <3.5	0.835 (0.646–1.079)	0.168	
≥3.5	0.691 (0.556–0.858)	**<0.001**	
Smoking history (n, %)			0.848
Current	0.925 (0.722–1.183)	0.533	
Past	0.714 (0.593–0.860)	**<0.001**	
Alcohol drinking history (drinks/week)			0.754
<1	0.886 (0.707–1.111)	0.296	
1–4	0.742 (0.600–0.916)	**0.006**	
>4	0.815 (0.509–1.306)	0.396	
BMI (kg/m^2^)			0.747
<18.5	0.910 (0.753–1.100)	0.329	
≥18.5, <25	1.067 (0.632–1.801)	0.808	
≥25, <30	0.721 (0.584–0.891)	**0.002**	
≥30	1.126 (0.721–1.760)	0.602	
Hypertension			0.695
Yes	0.804 (0.651–0.993)	0.043	
No	0.752 (0.621–0.911)	**0.004**	
Diabetes			0.946
Yes	1.045 (0.749–1.459)	0.796	
No	0.748 (0.644–0.870)	**<0.001**	
CVD			0.350
Yes	1.110 (0.625–1.850)	0.730	
No	0.780 (0.628–0.928)	**0.009**	

Full adjustment: adjusted for all variables such as age, race, educational level, PIR, smoking history, alcohol drinking history, hypertension, diabetes, cardiovascular disease, excluding subgroup-specific factors. Bonferroni correction was applied to adjust the significance threshold for both the subgroup *P*-values and the *P* for interaction; bold values in the table body indicate statistical significance after adjustment.

Consistent inverse associations were also noted in several other subpopulations, including non-Hispanic white (OR = 0.751, 95% CI: 0.617 – 0.915, *P* = 0.004), individuals with a college or university education (OR = 0.716, 95% CI: 0.595–0.861, *P* = 0.001), and those with a PIR ≥ 3.5 (OR = 0.691, 95% CI: 0.556–0.858, *P* < 0.001). Former smokers (OR = 0.714, 95% CI: 0.593–0.860, *P* < 0.001), moderate drinkers (1–4 drinks/week, OR = 0.742, 95% CI: 0.600–0.916, *P* = 0.006), and participants without hypertension (OR = 0.752, 95% CI: 0.621–0.911, *P* = 0.004), diabetes (OR = 0.748, 95% CI: 0.644–0.870, *P* < 0.001), or cardiovascular disease (OR = 0.780, 95% CI: 0.628–0.928, *P* = 0.009) also showed significant associations.

No significant interactions were observed across other subgroups (all *P* for interaction > Bonferroni-corrected threshold).

## Discussion

In our analysis of the total population, no statistically significant association was found between ALI and the presence of KSD. To explore whether this relationship varied by sex, we conducted a stratified analysis. The results revealed a significant association in males, whereas no meaningful link was observed in females. Specifically, among female participants, the fully adjusted logistic regression model showed an odds ratio of 1.020 (95% CI: 0.944–1.101; *P* = 0.616), suggesting no independent association. Consistent with this, ROC analysis in the female group produced a modest AUC of 0.633, indicating limited predictive value. The smoothed curve also suggested a slight upward trend in risk, which contrasted with the inverse relationship identified in the male subgroup. These observations suggest that ALI may function differently between sexes in its association with kidney stone risk. Accordingly, we focused subsequent analyses on males, in whom the association remained consistent after adjusting for potential confounders.

A number of biological and immunological factors may help explain the observed sex-specific differences in the predictive role of ALI. Given that ALI incorporates serum albumin, BMI, and NLR, physiological disparities in these parameters could influence its behavior across sexes. Males typically have greater muscle mass, higher BMI, and elevated albumin levels, which together may elevate ALI scores and strengthen their association with inflammatory or metabolic outcomes (20). In contrast, estrogen in females exerts anti-inflammatory effects by modulating oxidative stress and cytokine signaling, potentially diminishing the link between inflammation and stone development (21, 22). Moreover, immune response profiles–including neutrophil and lymphocyte counts–vary between sexes, which could further contribute to these differences. Variability in urinary composition and known sex-based differences in stone-forming risk factors also support the need for stratified interpretation (23, 24).

The results from this male subgroup revealed a significant inverse association between ALI and KSD prevalence. Specifically, we found that each unit increase in ALI was associated with a 22.7% reduction in kidney stone risk (OR = 0.773, 95% CI: 0.675–0.885, *P* < 0.001). These findings suggest that ALI may serve as an independent protective factor against KSD in males, likely due to its composite role in reflecting both inflammatory and nutritional status, which are important in the pathogenesis of kidney stones.

Advanced Lung Cancer Inflammation Index is a well-established prognostic marker in conditions influenced by systemic inflammation and nutritional status, such as cancer and chronic diseases. It has shown strong predictive value for survival outcomes in lung cancer and has been associated with mortality risk in T2DM, hypertension, liver cancer, and heart failure (12–15, 25, 26). In CKD, a condition marked by progressive renal dysfunction, systemic inflammation, and malnutrition, higher ALI levels have been inversely linked to disease prevalence, suggesting a potential protective role in renal health (16). Unlike chronic kidney disease, which primarily involves progressive renal dysfunction, KSD pathogenesis is largely driven by urinary supersaturation and crystallization. To our knowledge, our study is the first to evaluate ALI as a potential predictor of nephrolithiasis risk, thus expanding its clinical utility beyond its traditional applications.

Advanced Lung Cancer Inflammation Index is calculated by multiplying BMI by serum albumin and dividing by NLR. Unlike single inflammatory markers, ALI provides a holistic assessment of systemic health by incorporating both nutritional and inflammatory parameters. The observed inverse association between ALI and KSD likely reflects the intricate interplay of inflammation, oxidative stress, and metabolic disturbances, all of which contribute to kidney stone pathogenesis.

Neutrophil-to-lymphocyte ratio, a well-established marker of systemic inflammation, has been associated with an increased risk of kidney stone disease (27, 28). Neutrophils contribute to inflammation by releasing pro-inflammatory cytokines such as IL-8, IL-6, and neutrophil elastase, while lymphocytes secrete interferon-γ and tumor necrosis factor-α (TNF-α) (29). These cytokines, particularly IL-6 and TNF-α, disrupt calcium and oxalate metabolism, leading to increased urinary supersaturation and promoting crystal formation. Additionally, inflammation-induced oxidative stress may further exacerbate renal tubular injury, creating a favorable environment for stone formation (30–32).

Body mass index, a key component of ALI, has been identified as a significant risk factor for KSD recurrence (33). A meta-analysis of 13 cohort studies demonstrated that for every 5-unit increase in BMI, the relative risk of kidney stone formation increased by 21% (RR = 1.21, 95% CI: 1.12–1.30) (34).

Obesity contributes to KSD pathogenesis primarily by altering urinary composition. Increased body weight is associated with reduced urinary citrate and ammonium excretion, leading to urine acidification and promoting uric acid crystallization (35). Additionally, obesity increases urinary excretion of uric acid, oxalate, sodium, and phosphate, all of which are risk factors for calcium oxalate stone formation (36, 37). Metabolic abnormalities associated with obesity, such as insulin resistance and hyperinsulinemia, further contribute to KSD (38). Insulin resistance disrupts renal ammoniagenesis and Na^+^/H^+^ exchange, fostering an acidic urinary environment that facilitates stone formation (39, 40). Moreover, hyperinsulinemia reduces urinary citrate excretion while increasing urinary calcium, uric acid, and oxalate levels, thereby exacerbating stone risk (41, 42).

Serum albumin, another critical component of ALI, plays a pivotal role in maintaining nutritional status and oncotic pressure. Hypoalbuminemia is a well-recognized marker of chronic systemic inflammation and a negative acute-phase reactant (43). Inflammatory conditions enhance vascular permeability, leading to albumin leakage and subsequent hypoalbuminemia (44). Given albumin’s role in binding calcium and preventing calcium salt precipitation, lower serum albumin levels may increase urinary excretion of free calcium and other lithogenic substances, thereby promoting stone formation.

Overall, ALI, which combines indicators of nutritional condition (serum albumin and BMI) with systemic inflammatory status (NLR), reflects a broader picture of physiological health and homeostasis. Lower ALI values are indicative of a pro-inflammatory state accompanied by nutritional depletion and metabolic imbalance–all of which have been implicated in kidney stone development (27, 33, 38). Chronic inflammation can interfere with calcium and oxalate regulation, heighten oxidative stress, and injure renal epithelial cells, thereby increasing the likelihood of lithogenesis (29–32). Concurrently, metabolic abnormalities such as insulin resistance and acidic urinary pH create a biochemical milieu that favors stone formation (38–40). Moreover, reductions in serum albumin and body mass index may increase the urinary excretion of lithogenic compounds, contributing to a higher risk of stone formation (33, 43, 44). Taken together, these mechanisms may explain the inverse relationship between ALI and kidney stone risk observed in our study.

Building on these mechanistic insights, our study provides further evidence supporting the role of ALI in kidney stone disease. We observed an inverse association between ALI and KSD, suggesting that higher ALI may be protective against kidney stone formation. Our subgroup analysis revealed that this inverse association was most prominent among older adults (60–80 years), overweight individuals (BMI 25–29.9), and those without comorbidities such as hypertension, diabetes, or cardiovascular disease. These findings imply that ALI, a composite index that incorporates both nutritional (BMI, albumin) and inflammatory (NLR) components, may serve as a more sensitive and comprehensive predictor of kidney stone risk, particularly in metabolically healthier individuals.

Prior studies have established that inflammation plays a critical role in the pathogenesis of kidney stones by promoting oxidative stress, renal tubular injury, and crystal retention (45). In our study, individuals with higher ALI, particularly those in older age groups, likely benefit from better nutritional reserves and reduced systemic inflammation, both of which contribute to a less lithogenic environment and lower risk of kidney stone formation (46). Interestingly, moderately elevated BMI has been associated with reduced metabolic risk in certain chronic conditions, an effect often referred to as the “obesity paradox” (47). Therefore, in our study, individuals with a higher ALI in the overweight subgroup may experience a favorable balance between nutritional status and inflammatory markers, further supporting the protective effect of ALI against KSD.

Furthermore, ALI could serve as a simple, cost-effective screening tool for identifying high-risk individuals, guiding targeted dietary interventions to reduce stone risk. Additionally, emerging markers like Butyrylcholinesterase (BuChE) have been explored in post-surgical inflammation and may hold potential in KSD risk assessment. Advances in digital health, such as the Internet of Things (IoT), are also transforming disease monitoring and prevention. While these aspects extend beyond our study, they offer promising directions for future research (48, 49).

This study has several limitations that warrant consideration. First, the cross-sectional design of the NHANES dataset limits our ability to infer causal relationships. Second, reliance on self-reported data may introduce recall or reporting bias, and the absence of certain confounding variables may affect the robustness of the associations observed. Third, our approach to handling missing data involved complete-case analysis, which excluded participants with any missing values. This strategy, while straightforward, may have introduced selection bias if the data were not missing completely at random. Although more sophisticated techniques such as multiple imputation were contemplated, they were ultimately not applied due to the complexity and structure of missingness in the dataset.

The relatively small sample size, especially in certain subgroups, may have limited the statistical power to detect significant associations. These limitations highlight the need to validate our findings in broader and more diverse populations. In particular, the lack of a significant association in females–despite suggestive trends–calls for larger, longitudinal studies to clarify sex-specific effects. In addition to population-level validation, future studies should investigate the biological mechanisms linking ALI and kidney stone formation, potentially through *in vivo* or *in vitro* models. Moreover, the role of ALI in predictive modeling and clinical risk stratification deserves further exploration, especially given its simplicity and accessibility as a composite index reflecting inflammation and nutritional status.

## Conclusion

Advanced Lung Cancer Inflammation Index scores were independently associated with a reduced risk of kidney stones, especially among men without metabolic diseases. Because ALI is a simple measure of inflammation and nutrition, it may be useful in clinical settings to identify people at higher risk. However, this study used cross-sectional data, so we cannot be sure if ALI directly reduces kidney stone risk. Future prospective studies should confirm these findings and evaluate the usefulness of ALI for predicting and preventing kidney stones.

## Data Availability

The original contributions presented in this study are included in this article/Supplementary material, further inquiries can be directed to the corresponding author.
