# Supplementary figures and images for: The advanced lung cancer inflammation index as a predictor of kidney stone risk in men: a cross-sectional analysis
Source: Front Nutr. 2025 Jul 24;12:1568427. doi: 10.3389/fnut.2025.1568427 (PMC12329308; doi:10.3389/fnut.2025.1568427)

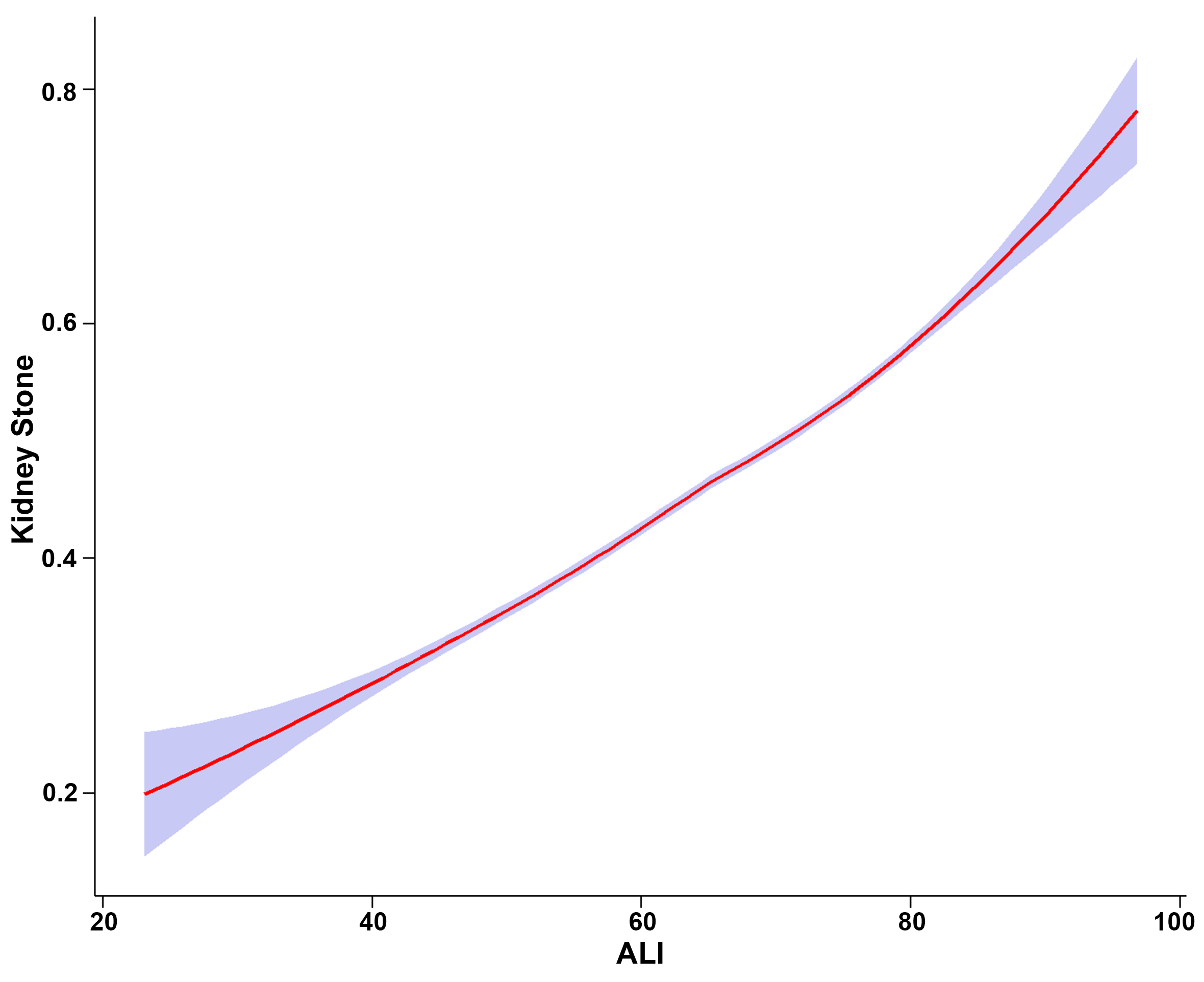

Supplement: Supplementary file 1 [file Image_1.tif]

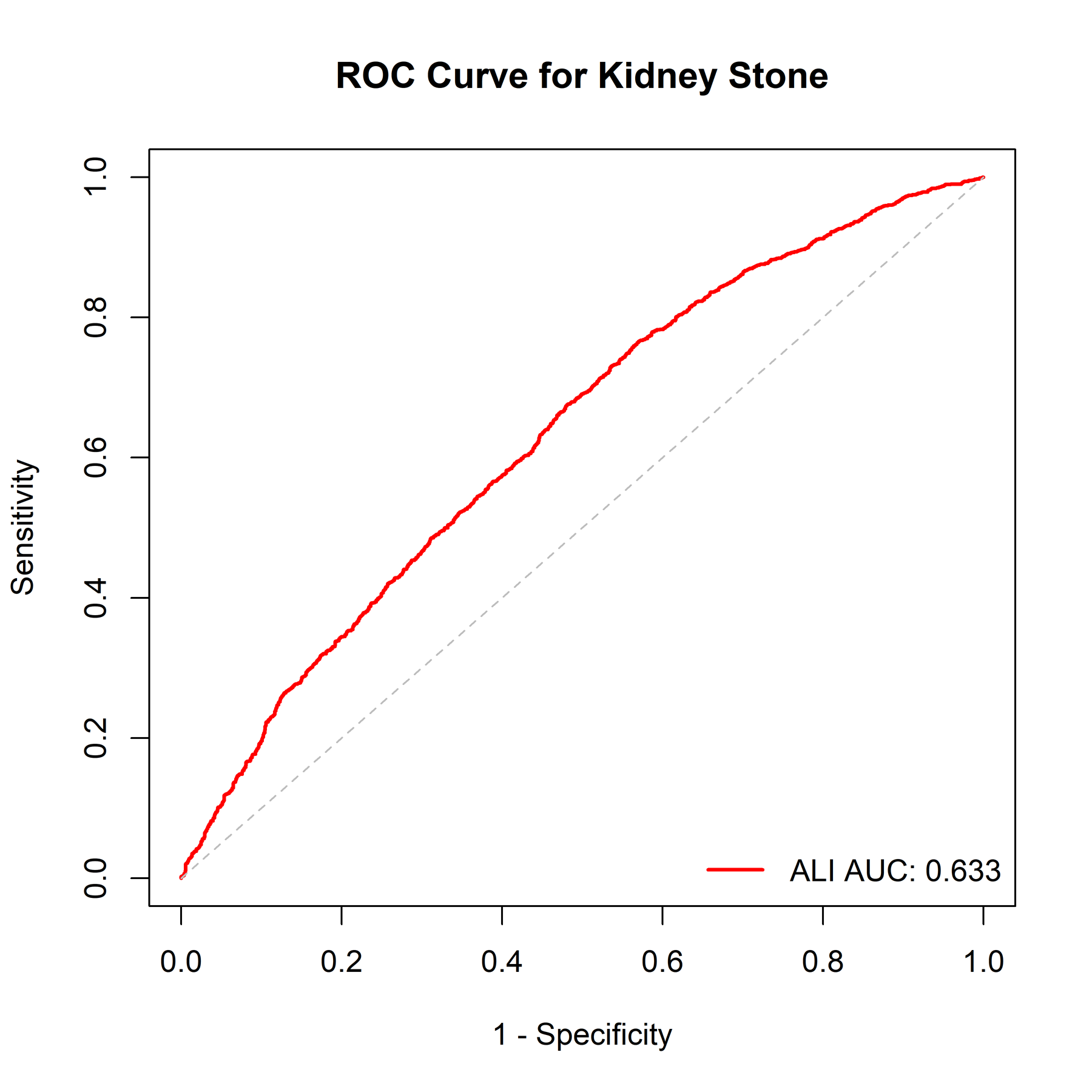

Supplement: Supplementary file 2 [file Image_2.tif]
