# Supplementary material for: The advanced lung cancer inflammation index as a predictor of kidney stone risk in men: a cross-sectional analysis
Source: Front Nutr. 2025 Jul 24;12:1568427. doi: 10.3389/fnut.2025.1568427 (PMC12329308; doi:10.3389/fnut.2025.1568427)
Supplement: Supplementary file 3 [file Table_1.docx]

**Supplementary Table 1** **Weighted logistic analysis of ALI and kidney stone risk in females.**

| **Model** | **ALI (Continuous variable), OR (95% CI), P value** | **Quartile 1 (Reference)** | **Quartile 2, OR (95% CI), P value** | **Quartile 3, OR (95% CI), P value** | **Quartile 4, OR (95% CI), P value** |
| --- | --- | --- | --- | --- | --- |
| Model 1 | 1.063  (1.047–1.079), **P<0.001** | Reference | 1.166  (0.452–3.008), P = 0.752 | 1.655  (0.678–4.041), P= 0.268 | 2.845 (1.173–6.900),  **P = 0.021** |
| Model 2 | 1.059  (1.042–1.076), **P<0.001** | Reference | 1.080 (0.416–2.808), P = 0.874 | 1.387 (0.562–3.424),  P = 0.478 | 2.284 (0.930–5.613),  P = 0.072 |
| Model 3 | 1.020 (0.944–1.101), P=0.616 | Reference | 0.953  (0.367–2.477), P = 0.921 | 1.032 (0.413–2.579),  P = 0.947 | 1.147  (0.434–3.035),  P = 0.782 |

OR, odds ratio; 95% CI, 95% confidence interval.

Model 1, no covariates were adjusted.

Model 2, adjusted for age, race, educational level, and PIR.

Model 3, adjusted for age, race, educational level, PIR, smoking history, alcohol

drinking history, hypertension, diabetes, cardiovascular disease.

Bold values in the table body indicate statistical significance at P < 0.05.
